# Screening of *Myo7A* Mutations in Iranian Patients with Autosomal Recessive Hearing Loss from West of Iran

**Published:** 2017-01

**Authors:** Samira ASGHARZADE, Somayeh REIISI, Mohammad Amin TABATABAIEFAR, Morteza HASHEMZADEH CHALESHTORI

**Affiliations:** 1. Dept. of Molecular Medicine, School of Advanced Technologies in Medicine, Tehran University of Medical Sciences, Tehran, Iran; 2. Cellular and Molecular Research Center, Shahrekord University of Medical Sciences, Shahrekord, Iran; 3. Dept. of Genetics, Faculty of Basic Sciences, University of Shahrekord, Shahrekord, Iran; 4. Dept. of Genetics and Molecular Biology, School of Medicine, Isfahan University of Medical Sciences, Isfahan, Iran

**Keywords:** *MYO7A*, Deafness, Linkage analysis, DFNB2, Iran

## Abstract

**Background:**

Hearing loss (HL) is the most frequent neurosensory impairment. HL is highly heterogeneous defect. This disorder affects 1 out of 500 newborns. This study aimed to determine the role of DFNB2 locus and frequency of *MYO7A* gene mutations in a population from west of Iran.

**Methods:**

Thirty families investigated in Shahrekord University of Medical Sciences in 2014, genetic linkage analysis via four short tandem repeat markers linked to *MYO7A* was performed for two consanguineous families originating from Hamedan (family-13) and Chaharmahal-Bakhtiari (family-32) provinces of Iran, co-segregating autosomal recessive HL and showed no mutation in *GJB2* gene in our preliminary investigation. All 49 coding exons and exon- intron boundaries of the *MYO7A* gene were amplified by PCR and analyzed using direct DNA sequencing.

**Results:**

Two of families displayed linkage to DFNB2. Family-13 segregated a homozygous missense mutation (c.6487G>A) in exon 48 that results in a p.G2163S amino acid substitution in C-terminal domain of the myosin VIIA protein. While family-32 segregated a homozygous nonsense mutation (c.448 C>T) in exon five, resulting in a premature truncation at amino acid position 150 (p.Arg150X) in the motor domain of this protein.

**Conclusion:**

Mutation frequency of *MYO7A* gene in different populations of Iran as well as cause of HL in most cases are still unknown and more extensive studies have to be done.

## Introduction

Hereditary non-syndromic hearing loss (HL) is the most common defect that attributed to both genetical and environmental factors and has an incidence of 1 in 500 infants (1). HL is extremely heterogeneous defect and up to date, about 62 genes have been identified for autosomal recessive non-syndromic hearing loss (ARNSHL) (2). More than thousands mutations in more than 60 genes have been identified which associate with HL (2), however, most of HL causes and their mutation is still unknown. One group of genes associated with HL is Myosin. Myosins are a superfamily consisting of more than 35 classes and they are actin-based molecular motors (3). N-terminal region of myosin heavy chain consists of highly conserved sequences and its head domain following a divergent neck domain. N-terminal region of head domain binds to actin and ATP (4). C-terminal region of Myosin is known as tail domain and in various classes of Myosin is highly divergent. Myosins are mainly classified to conventional and unconventional (5). Myosin VIIA (*MYO7A*) belongs to unconventional myosins, has 49 exons, and mostly is expressed in inner ear and retina (6). *MYO7A* is made in inner eye and contribute to organization of the stereocilia in the hair bundles.

Up to now, 340 different mutations and more than 248 protein variants in *MYO7A* associated with these disorders have been reported (7) (http://www.umd.be-/*MYO7A*/) that affect the head and tail domains (7). Defects in *MYO7A* are cause of syndromic (USH1B) and nonsyndromic deafness (two form of dominant (DFNA11) and recessive (DFNB2) deafness) (7, 8). Mutations in the head domain are thought to affect *MYO7A* motor function, but there is little known about the affect protein function when mutations in the tail domain (8). This present study aimed to determine mutation frequency in *MYO7A* and linkage analysis of DFNB2 locus.

## Materials and Methods

### Subjects and Sampling

The Institutional Review Boards of Tehran University of Medical Sciences and Shahrekord University of Medical Sciences approved this descriptive laboratory study in 2014.

Thirty families (405 individual) including 158 affected individuals of clients of state welfare organization and exceptional children education organization from Hamedan and Kohgiluyeh and Boyer-Ahmad provinces in the west of Iran were included in this study. Families investigated had at least two affected children. Parents were unaffected and majority of them had consanguineous marriages. All patients underwent audiogram hearing test and ear, nose and throat (ENT) specialist confirmed presence of HL in patients. All families were informed and written consent was obtained. Then 5 cc peripheral blood in tubes containing 0.5 M EDTA from all available members of all families were captured.

### DNA extraction

Genomic DNA was extracted from blood samples using phenol-chloroform standard method (9). DNA quality (UNICO 2100, USA) and on 0.5% agarose gel stained with 0.5 x gel red (10).

### STR (Short tandem repeats) marker genotyping and linkage analysis

For analyzing of locus, four different STR markers were used. Upon encountering an uninformative marker, further markers were examined. In Table 1, used markers and their characteristics are shown.

**Table 1:** STR marker used in study and their characteristics

**STR marker **	**Physical position **	**HZ (%) **	**Amplicon Size **	**Forward Primer **	**Reverse Primer **
D11S4179	76,396,260 –76,396,495	72	200–256	GGATGTAAGAGTAACTGGCTCCG	GAAAATGTTCTGCCTGAGGG
D11S4186	76,968,518 –76,968,685	79	154–175	ATTCTCCCAATCTATCGCTC	GGGCAGTAATGATGATGTG
D11S4079	77,119,447 –77,119,701	75	217–265	CAGCAAGATCCTGTCTCAA	CTCCTTAAAGTGGGGGAGTT
D11S911	77,448,583 – 77,448,769	85	159–203	CTTCTCATGCTTGACCATTT	CTTCTGAACAATTGCCACAT

HZ: Heterozygous

The criteria for selecting these markers are as following greater heterozygosity values, shorter fragments in length and lying close to the known locus.

For S-Link and LOD score calculation, we used genetic software Easy linkage plus ver. 5.05 (11). For S-Link calculation, we used Fast Slink ver. 2.51. Two-point and multipoint parametric LOD scores were calculated by Super link ver. 1.6, Gene hunter ver. 2.91, respectively. For LOD score calculations using this software, inheritance pattern of autosomal recessive, complete penetrance and disease allele frequency of 0.001 were assumed. Haplopainter ver. 029.5 software was used for reconstruction of haplotypes (12). Negative subjects for connexin 26 gene mutation are selected for linkage analysis. STR markers were selected based on their physical distance found at NCBI Uni STS. For markers Primers, NCBI Map Viewer is used and Touchdown program is used for markers.

### Mutation screening of MYO7A

In order to analyze the mutation in families linked to mentioned locus, for 49 exons, intronexon boundaries, primers were designed by Oligo ver. 6.7.1.0 (National Biosciences Inc., Plymouth, MN, USA) and Primer3 software (https://www.genome.com/cgi-bin/primer/primer3_www.cgi). Thermal cycling conditions for amplifying exons were as follows: 95 °C for primary denaturation for 5 min, 8 touchdown cycles of 94 °C denaturation of DNA strands for 30 sec, 55 °C to 62 °C annealing temperature for 30 sec, 72 °C extension for 30 sec and in next 25 cycles of 94 °C denaturation for 30 sec, 54 °C annealing temperature for 30 sec, 72 °C extension for 30 sec and 72 °C final extension for 5 min. In each PCR, 0.5 μl of each primers (10 PM), 0.5 μl dNTP mix (10 mM), 1 μl MgCl_2_ (50 mM), 0.1 μl Taq polymerase (5 unit/μl), 2 μl genomic DNA (50 ng). The total volume was adjusted to 25 μl by ddH2O. After amplifying desired piece of DNA, PCR product for each family separately was run in 8–12% polyacrylamide gel electrophoresis at 35–40 mA for 3 h, following silver staining of the gel, bands were visualized. DNA sequencing of the PCR- products was performed bi-directionally on an ABI 3730XL automated sequencer (Applied Bio-systems, Macrogen and, Seoul, Korea) than any variant in this gene sequence using Seqmqn software analyzer and Chromas software http://www.technelysium.com.au/chromas.html were detected.

## Results

Majority of studied subjects showed severe to profound bilateral neurosensory HL. Seventy percent of investigated families in present study were consanguineous families. Based on information given in pedigree, type of HL was autosomal recessive. Among the investigated families, two families displayed linkage to DNFB2 locus and by analysis of *MYO7A* gene two mutations have been detected. Estimated maximum Slink for these 30 families ranged from 1.12 to 7.68, two-point and multipoint LOD and S-link scores related to two families are indicated in Table 2.

**Table 2:** S-Link and LOD scores calculated for family linked to DFNB3

**Family**	**S-LINK**	**Two point LOD score**	**Multipoint LOD score**
Family- 13	2.53	1.96	2.30
Family -32	2.53	3.2	1.3

**Fig. 1:**
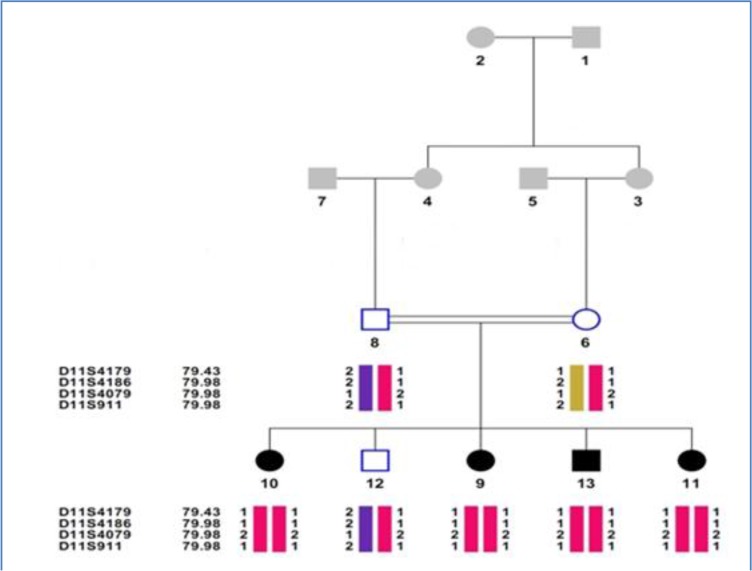
pedigree and Haplotype of Family-13

**Fig. 2:**
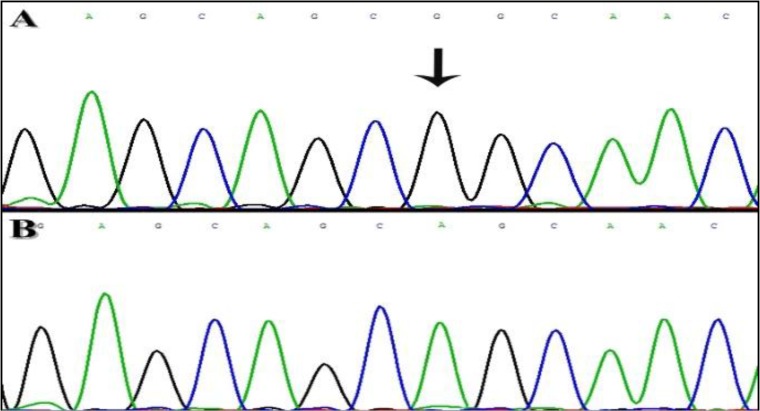
Chromatograms results of *MYO7A* mutation in Family-13. A: normal alleles for c.6487 G>A. B: mutant allele of c.6487 G>A.

Family-13: This family was from Hamedan province (Fig. 1) and after sequencing of *MYO7A* gene, homozygous missense mutation c.6487 G>A was found in exon 48 that result in an amino acid substitution (p.G2163S) in **C-terminal** FERM (F for 4.1 protein, E for ezrin, R for radixin and M for moesin) domain of the myosin VIIA protein (Fig. 2). This Mutation in parents and non-affected children was in heterozygous state and parents had consanguineous marriage. Onset of hearing loss was prelingual and the progress of HL was unchanged, audiogram-hearing test confirmed the type of severe to profound.

**Fig. 3:**
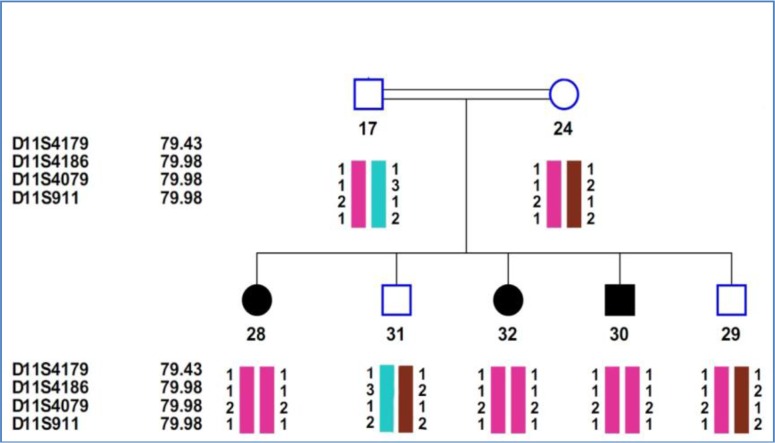
Pedigree and Haplotype of Family-32

Family-32: This family was from Kohgiluyeh and Boyer-Ahmad province and consanguineous marriage was three grade (Fig. 3).

**Fig. 4:**
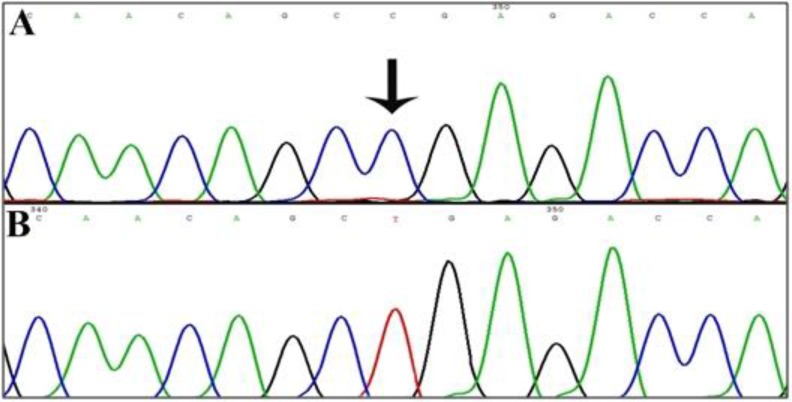
Chromatograms results of *MYO7A* mutation in Family-32. A: normal alleles for c.448 C>T. B: mutant allele of c.448 C>T.

Affected individuals in this family showed profound hearing loss along with progressive retinitis pigmentosa and vestibular dysfunction. Mentioned clinical signs and symptoms may suggest presence of USHB1. By sequencing of *MYO7A* gene, homozygous nonsense mutation (c.448 C>T) in exon five was detected (Fig. 4) that resulting in a premature truncation at amino acid position 150 (p.Arg150X) in the motor domain of this protein.

## Discussion

In this study, 30 families with at least two affected subjects from provinces of Hamedan and Kohgiluyeh and Boyer-Ahmad were investigated. Linkage analysis and haplotype analysis of STR molecular markers in DFNB2 locus for families with no mutation in connexin 26 were carried out. Since mutation in connexin 26 is responsible for 18.29% of hearing loss in consanguineous families (13) and hearing loss is highly heterogeneous and due to presence of different ethnic groups (14) and high rate of consanguineous marriages in Iran, analysis of other loci associated with hearing loss in different populations of Iran is necessary (15–17).

Thus, by analysis of families with no mutation in connexin 26 and other genes associated with HL. We can gain new insight into frequency of these genes. In this investigation, two families were found to be linked to DFNB2 locus. After sequencing of *MYO7A* gene, 2 variants c.448C>T in Motor domain and c.6487 G>A in the C-terminal FERM_2_ domain of *MYO7A* have been identified.

*MYO7A* gene (NM_000260.3) is consisting of 49 exons that encode a protein with 2215 amino acids. Up to date, majority of mutations identified in *MYO7A* were associated with ARNSHL and Usher syndrome and few number of these mutations were associated with ADNSHL (DFNB11) (6). These mutations mostly cause loss of protein function. DFNB2 locus in one Tunisian family with non-syndromic hearing loss was mapped (18), and up to date mutations in *MYO7A* in patients with hearing loss from different populations such as China (19), Tunisia (20), Pakistan (21), Taiwan (22) and Iran have been reported (23). One of identified mutations in this study is c.448C>T which is nonsense and changes argi-nine amino acid in protein at position 150 for stop codon (p.Arg150X), this mutation occurs in motor domain of Myosin VII protein. The motor domain contains actin- binding domain and thought to be involved in mechanical movement of hair bundles using ATP, mutation in this protein binding site results in loss of function of actin protein and as a result, action cannot bind to the site and consequently cannot have normal movements. This mutation was first reported in patients with Usher syndrome that caused an abnormal protein near ATP binding site (24, 25). Based on clinical examination of patients, this study may suggest the presence of Usher syndrome type I.

Mutation c.6487 G>A converts glycine amino acid to serine at position 2163 (Gly2163Ser). This mutation occurs in C-terminal FERM_2_ domain of protein interacts with specific proteins and confers specificity to Myosin VIIA and mutations in this domain results in protein instability and impairment in binding of other proteins to tail domain. This mutation was first in one Palestinian family and then later in one Iranian family was reported (21, 26). After sequencing of *MYO7A* gene, this mutation was attributed to cause of hearing loss in family-13.

Mutation frequency of *MYO7A* gene in different populations of Iran as well as cause of hearing loss in most cases are still unknown and more extensive studies have to be done.

## Conclusion

The mutation analysis of *MYO7A* gene led to the identification of two mutations in two families including missense mutations in different regions of the myosin-XV protein. *MYO7A* mutational frequency displayed of 6.67% in our cohort of deaf Iranian families.

## Ethical considerations

Ethical issues (Including plagiarism, informed consent, misconduct, data fabrication and/or falsification, double publication and/or submission, redundancy, etc.) have been completely observed by the authors.
